# The impact of an open or laparoscopic approach on the development of metachronous peritoneal metastases after primary resection of colorectal cancer: results from a population-based cohort study

**DOI:** 10.1007/s00464-022-09041-z

**Published:** 2022-01-20

**Authors:** Robin J. Lurvink, Anouk Rijken, Checca Bakkers, Valery E. Lemmens, Philip R. de Reuver, Jurriaan B. Tuynman, Niels F. Kok, Simon W. Nienhuijs, Felice N. van Erning, Ignace H. J. T. de Hingh

**Affiliations:** 1 Department of Surgery, Catharina Cancer Institute, Catharina Hospital, PO Box 1350, 5602 ZA Eindhoven, The Netherlands; 2 Department of Research and Development, Netherlands Comprehensive Cancer Organization, Utrecht, The Netherlands; 3 Department of Surgery, Radboud University Medical Center, Nijmegen, The Netherlands; 4 Department of Surgery, Amsterdam University Medical Centers, location VUMC, Amsterdam, The Netherlands; 5 Department of Surgery, Netherlands Cancer Institute, Amsterdam, The Netherlands; 6 GROW – School for Oncology and Developmental Biology, Maastricht University, Maastricht, the Netherlands

**Keywords:** Colorectal cancer, Peritoneal metastases, Surgical approach, Open surgery, Laparoscopic surgery

## Abstract

**Background:**

This study aimed to assess the impact of open or laparoscopic resection of primary colorectal cancer (CRC) on the development of metachronous colorectal peritoneal metastases (CPM) in a population-based cohort.

**Materials and methods:**

This was a retrospective, population-based study of CRC patients who underwent open or laparoscopic resection of the primary tumour in the Netherlands between January 1st and June 30th 2015. Patients with synchronous metastases were excluded. CPM were considered metachronous if diagnosed ≥ 90 days after resection of primary CRC. Multivariable cox regression analysis was performed to correct for tumour location, histology, differentiation, and stage, nodal stage, tumour perforation, primary surgery type, and unclear resection margins.

**Results:**

In total, 1516 CRC patients underwent open resection and 3236 CRC patients underwent laparoscopic resection, with a 3-year cumulative incidence of metachronous CPM of 7.3% and 3.7%, respectively (*p* < 0.001), after median follow-up of 42 months. Open surgical approach was significantly associated with the development of metachronous CPM: HR 1.4 [95%CI 1.1–1.8]. Other prognostic factors were mucinous adenocarcinoma histology (HR 1.6, 95%CI 1.0–2.5), T4 stage (HR 3.2, 95%CI 2.3–4.5), N1 stage (HR 2.9, 95%CI 2.1–4.0), and N2 stage (HR 4.2, 95%CI 2.9–6.1).

**Conclusions:**

Patients treated with open resection had a significantly higher risk to develop metachronous CPM than patients treated with laparoscopic resection. The mechanisms underlying this phenomenon remain unknown but might be related to differences in per-operative specimen handling, tumour spill, surgical trauma and pro-inflammatory response. This finding might imply the need for a personalized follow-up after primary resection of CRC.

Colorectal cancer (CRC) is the second most prevalent cancer worldwide, with an incidence of nearly two million patients in 2020 [1]. Despite the improvement of curative treatment options, recurrent disease occurs frequently. In CRC, the peritoneum is the second most prevalent metastatic site, after the liver [2–4].

Considering that curative-intent cytoreductive surgery for limited colorectal peritoneal metastases (CPM) is associated with a more favourable prognosis, timely detection of CPM is of utmost importance [5–7]. Unfortunately, CPM are difficult to detect on conventional imaging during normal follow-up and subsequently patients often present with advanced disease. Several factors, such as an advanced TNM stage at diagnosis, and mucinous or signet ring cell tumour histology have been found to be associated with an increased incidence of metachronous CPM. Thus, these parameters can be used to optimize follow-up for early detection of CPM [4].

In a previous population-based study we showed that synchronous CPM were less frequently diagnosed during laparoscopic resection than during open resection [8]. It was hypothesized that CPM might have been overlooked during laparoscopy due to an insufficient overview of the peritoneal cavity and the lack of tactile feedback. If this were true, this should result in an increased number of patients in whom peritoneal metastases are diagnosed during follow-up (i.e. metachronous CPM). A single-centre retrospective cohort study in patients with T4 colon cancer seemed to confirm this hypothesis, as they found a greater incidence of metachronous CPM after laparoscopic resection [9]. Such a finding could have serious consequences for the treatment of CRC, since laparoscopic resection has been increasingly applied given the lower complication rate, lower mortality rate, less major morbidity and a shorter hospital stay than open resection [10, 11].

Therefore, this study aimed to assess the impact of an open or laparoscopic approach on the incidence of metachronous peritoneal metastases in patients who underwent surgical treatment for CRC in a population-based cohort.

## Methods

### Data source

Data from the Netherlands Cancer Registry (NCR), which registers all newly diagnosed malignancies in the Netherlands, were used for this nationwide population-based cohort study. Trained data-managers routinely collect these data from hospital records. The International Classification of Disease – Oncology (ICD-O) was used to register the anatomical sites of the primary tumour and metastases, and the seventh edition of the Tumour Node Metastasis (TNM) classification was used to classify the tumour and nodal status. The clinical TNM stage was used when the pathological TNM stage was not available.

Normally, the NCR contains information on the primary tumour, metastases diagnosed at the time of diagnosis of the primary tumour, and primary treatment, after which a yearly update of the vital status is performed by linkage to the Dutch municipal administrative database. In 2019, the NCR data-managers performed a re-evaluation of all CRC patients diagnosed between January 1st 2015 and June 30th 2015, aiming for follow-up information on local or systemic recurrences and their treatment. All data were anonymized. No approval of a medical ethics committee was required.

### Patients and characteristics

All patients diagnosed with CRC between January 1st and June 30th 2015 in the Netherlands were included in the current study. If more than one primary colorectal tumour was diagnosed in the same patient, only the firstly diagnosed tumour was included, or, if simultaneously diagnosed, the tumour with the highest TNM stage was included. The location of the primary tumour was categorized into three anatomical subsites: (1) right-sided colon (C18.0, C18.2–18.4: cecum, ascending colon, hepatic flexure, transverse colon); (2) left-sided colon (C18.5–18.7: splenic flexure, descending colon and sigmoid); and (3) rectum (C19.9–20.9: rectosigmoid and rectum). Primary tumour histology was categorized into three subtypes: (1) adenocarcinoma (8000, 8010, 8020, 8140, 8144, 8210, 8211, 8220 8255, 8261, 8262, 8263, 8560); (2) mucinous adenocarcinoma (8480, 8481); and (3) signet ring cell carcinoma (8490).

Patients were excluded if they had a primary tumour located in the appendix, a neuroendocrine primary tumour, a non-adenocarcinoma tumour histology, or synchronous metastases. The following ICD-O codes were considered peritoneal metastases: C16.0–C16.9, C17.0–C17.9, C18.0–C18.9, C19.9, C20.9, C21.8, C23.9, C26.9, C48.0–C48.8, C49.4–C49.5, C52.9, C54.3–C54.9, C55.9, C56.9, C57.0–C57.8, C66.9, C67.0–C67.9, C76.2.

Among patients who underwent open or laparoscopic resection of primary CRC, follow-up data was used to assess the occurrence of metachronous peritoneal metastases (≥ 90 days after surgery for primary CRC). Patients in whom a laparoscopic resection was converted to open resection were considered to have undergone open resection.

### Statistical analyses

The 1- and 3-year cumulative incidence of metachronous CPM after open and laparoscopic resection of primary CRC was calculated considering death as competing event. Time to event was calculated from the date of surgery to the date of last follow-up (censor), diagnosis of metachronous CPM (event of interest), or death (competing event). The Gray’s test was used to compare differences in the cumulative incidence of metachronous CPM.

Baseline characteristics were compared between patients who underwent open or laparoscopic resection of primary CRC. Differences in continuous variables were compared with the unpaired t-test and presented as a mean (± standard deviation [SD]), and differences in categorical variables were compared using Chi-squared tests and presented as *n* (%). Missing data were excluded from comparative analyses.

Univariable cox regression analyses with death as competing event were performed to identify factors associated with the development of metachronous CPM. Time to event was calculated from the date of surgery to the date of last follow-up (censor), diagnosis of metachronous CPM (event of interest), or death (competing event). Variables with a *p* < 0.100 were combined in a multivariable cox regression model with respect to the number of patients developing metachronous CPM (10 events per degree of freedom) to prevent overfitting of the multivariable model. Dummy variables of missing data were included in the regression analyses.

All tests were two-sided and *p* < 0.050 was considered statistically significant. All analyses were performed using SAS statistical software (SAS system 9.4, SAS Institute, Cary, NC, United States).

## Results

### Study population

**Fig. 1:**
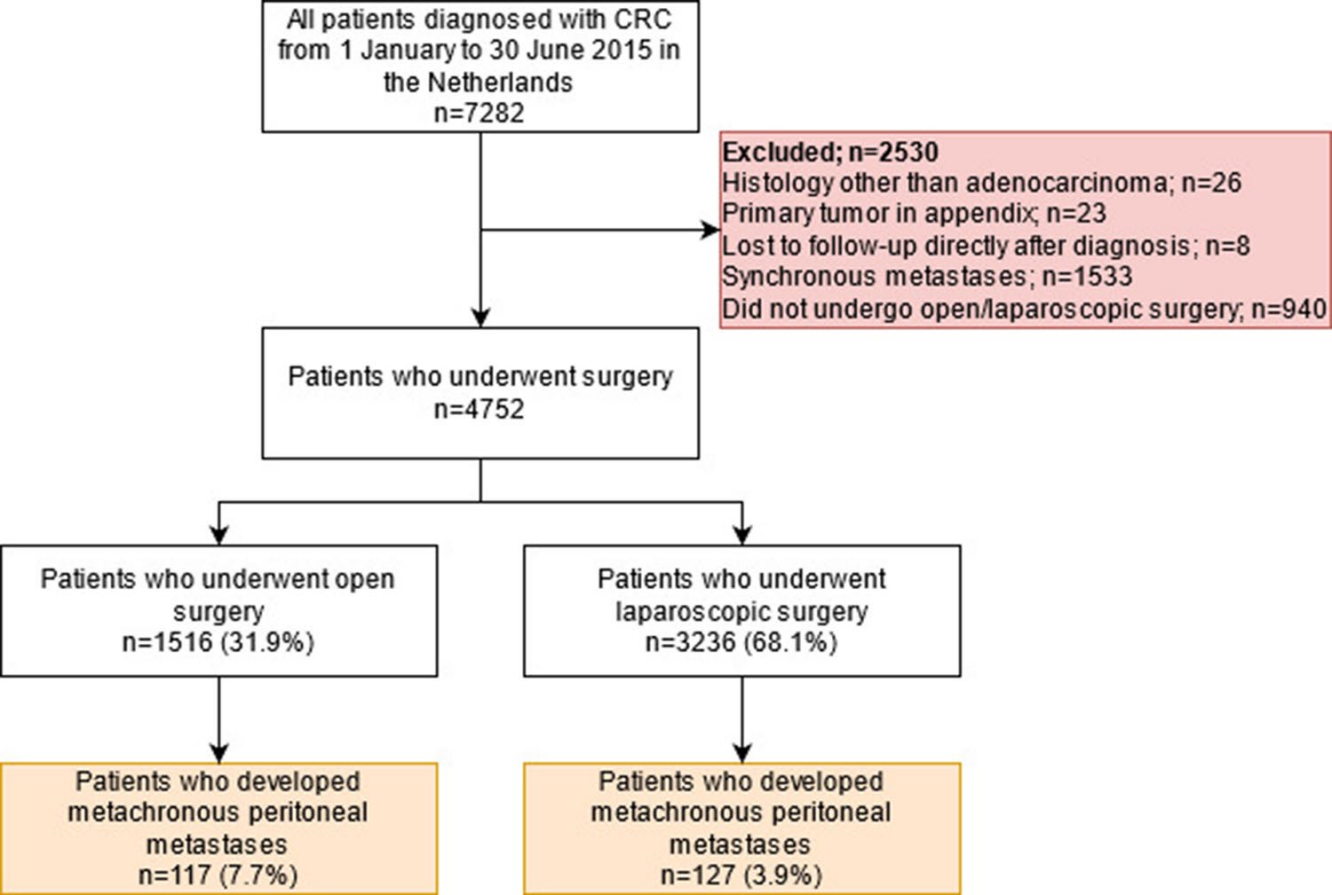
Study flowchart. *CRC* Colorectal cancer, *PM* Peritoneal metastases

The final study population comprised 4752 patients with CRC without synchronous metastases of whom 1516 underwent open resection (31.9%) and 3236 underwent laparoscopic resection (68.1%) of the primary CRC tumour (Fig. 1).

Table 1 contains an overview of the study population, stratified for surgical approach. Patients who underwent laparoscopic resection were younger, more often had a lower ASA classification, a primary tumour located in the rectum, an adenocarcinoma histology, good or moderate tumour differentiation, a T0-3 tumour stage, an N0 nodal stage, clear resection margins, and a non-perforated colon than patients who underwent open resection.

**Table 1:** Baseline characteristics

	Open surgery *N* = 1516	Laparoscopic surgery *N* = 3236	*P* value
Sex			0.551
Male	882 (58)	1853 (57)	
Female	634 (42)	1383 (43)	
Age	70 ± 11	68 ± 9	** < 0.001**
ASA			** < 0.001**
1	174 (11)	631 (20)	
2	746 (49)	1789 (55)	
3–4	388 (26)	517 (16)	
Missing data*	208 (14)	299 (9)	
Primary tumour location			** < 0.001**
Right colon	618 (41)	950 (29)	
Left colon	575 (38)	1220 (38)	
Rectum	323 (21)	1066 (33)	
Primary tumour histology			**0.006**
Adenocarcinoma	1345 (89)	2964 (92)	
Mucinous adenocarcinoma	155 (10)	248 (8)	
Signet ring cell carcinoma	16 (1)	24 (1)	
Primary tumour differentiation			** < 0.001**
Good/moderate	1179 (78)	2704 (84)	
Poor/none	160 (11)	218 (7)	
Missing data*	177 (12)	314 (10)	
Tumour stage			** < 0.001**
T0-3	1239 (82)	3013 (93)	
T4	276 (18)	222 (7)	
Missing data*	1 (0)	1 (0)	
Nodal stage			** < 0.001**
N0	933 (62)	2183 (67)	
N1	375 (25)	730 (23)	
N2	208 (14)	322 (10)	
Missing data*	0 (0)	1 (0)	
Colon perforation			** < 0.001**
No	1320 (87)	3068 (95)	
Yes	112 (7)	49 (2)	
Missing data*	84 (6)	119 (4)	
Resection margins			** < 0.001**
Not clear	54 (4)	52 (2)	
Clear	1446 (95)	3169 (98)	
Missing data	16 (1)	15 (0)	

*The category ‘missing data’ was not included in chi square analyses; Percentages might not add up to or exceed 100% due to rounding

### Metachronous colorectal peritoneal metastases

**Fig. 2:**
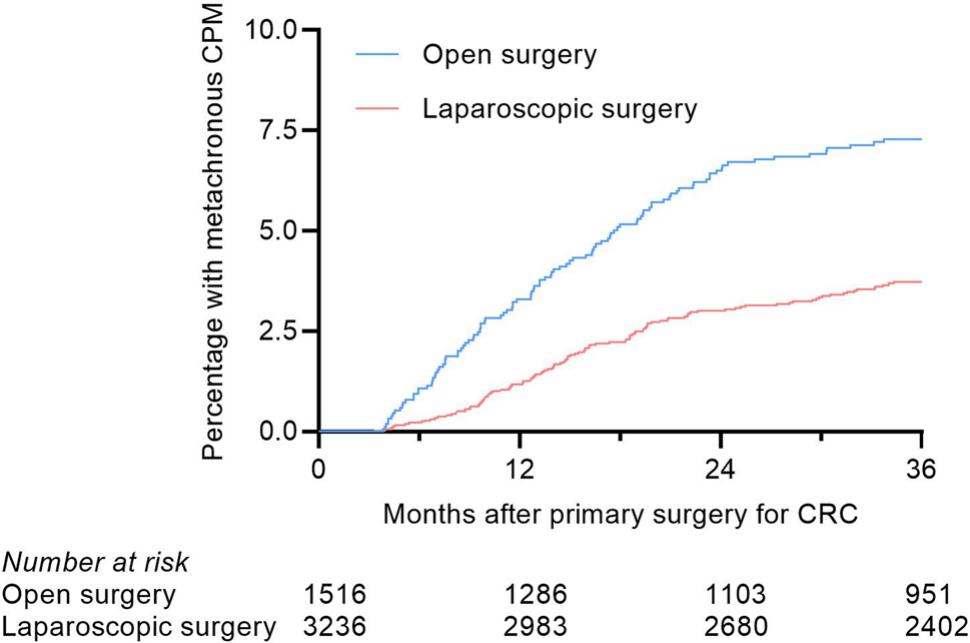
Cumulative incidence of metachronous peritoneal metastases after open or laparoscopic resection. *CPM* Colorectal peritoneal metastases, *CRC* Colorectal cancer

A total of 244 patients were diagnosed with metachronous CPM after a median follow-up of 42.4 months (interquartile range [IQR] 30.3–46.3). After open resection, 117 out of 1516 patients developed metachronous CPM, with a 1- and 3-year cumulative incidence of metachronous CPM of 3.3% (95% CI 2.5–4.3) and 7.3% (95% CI 6.1–8.7), respectively. After laparoscopic resection, 127 out of 3235 patients developed metachronous CPM, with a 1- and 3-year cumulative incidence of metachronous CPM of 1.2% (95% CI 0.8–1.6) and 3.7% (95% CI 3.1–4.5), respectively (*p* < 0.001) (Fig. 2).

In multivariable cox competing risk regression analysis (Table 2), a statistically significant association between open resection and the development of metachronous CPM (HR 1.4, 95% CI 1.1–1.8) was observed. Furthermore, the following factors were also associated with the development of metachronous CPM: histology of a mucinous adenocarcinoma (HR 1.6, 95% CI 1.0–2.5), T4 tumour stage (HR 3.2, 95% CI 2.3–4.5), N1 nodal stage (HR 2.9, 95% CI 2.1–4.0), and N2 nodal stage (HR 4.2, 95% CI 2.9–6.1).

**Table 2:** Cox competing risk analysis for the development of metachronous peritoneal metastases

Cox competing risk analysis	Metachronous PM	Univariable analysis			Multivariable analysis		
	N (%)	HR	95% CI	*P* value	HR	95% CI	*P* value
Primary surgery type				** < 0.001**			
Laparoscopic	127 (4)	Ref	Ref		Ref	Ref	Ref
Open	117 (8)	2.0	1.6–2.6		**1.4**	**1.1–1.8**	**0.016**
Sex							
Male	132 (5)	Ref	Ref		–	–	–
Female	112 (6)	1.2	0.9–1.5		–	–	–
Age				0.159			
< 50	16 (8)	1.6	0.9–2.7		–	–	–
50–74	158 (5)	Ref	Ref		–	–	–
≥ 75	70 (5)	1.1	0.9–1.50		–	–	–
ASA score				0.820			
ASA 1	40 (5)	1.0	0.7–1.4		–	–	–
ASA 2	128 (5)	Ref	Ref		–	–	–
ASA 3–6	46 (5)	1.1	0.8–1.5		–	–	–
Missing data	30 (6)	1.2	0.8–1.8		–	–	–
Primary tumour location				** < 0.001**			
Right colon	108 (7)	1.4	1.1–1.9		1.2	0.9–1.6	0.188
Left colon	87 (5)	Ref	Ref		Ref	Ref	Ref
Rectum	49 (4)	0.7	0.5–1.0		1.0	0.7–1.4	0.848
Tumour histology				** < 0.001**			
Adenocarcinoma	204 (5)	Ref	Ref		Ref	Ref	Ref
Mucinous adenocarcinoma	31 (8)	1.7	1.2–2.5		**1.6**	**1.0–2.5**	**0.042**
Signet ring cell carcinoma	9 (23)	5.3	2.7–10.5		2.3	0.9–5.4	0.053
Primary tumour differentiation				** < 0.001**			
Good/moderate	176 (5)	Ref	Ref		Ref	Ref	Ref
Poor/none	39 (10)	2.3	1.7–3.3		1.2	0.9–1.8	0.247
Missing data	29 (6)	1.3	0.9–2.0		0.8	0.5–1.3	0.329
Tumour stage				** < 0.001**			
T0–3	149 (4)	Ref	Ref		Ref	Ref	Ref
T4	95 (19)	5.9	4.5–7.6		**3.2**	**2.3–4.5**	** < 0.001**
Nodal stage				** < 0.001**			
N0	72 (2)	Ref	Ref		Ref	Ref	Ref
N1	91 (8**)**	3.5	2.6–4.8		**2.9**	**2.1–4.0**	** < 0.001**
N2	81 (15)	6.8	4.9–9.3		**4.2**	**2.9–6.1**	** < 0.001**
Tumour perforation				**0.008**			
No	215 (5)	Ref	Ref		Ref	Ref	Ref
Yes	17 (11)	2.2	1.3–3.6		1.0	0.5–1.8	0.960
Missing data	12 (6)	1.3	0.7–2.2		1.0	0.6–1.8	0.901
Resection margins				** < 0.001**			
Clear	223 (5)	Ref	Ref		Ref	Ref	Ref
Not clear	18 (17)	3.6	2.2–5.9		1.3	0.7–2.3	0.370
Missing data	3 (10)	2.2	0.7–7.0		0.8	0.2–2.6	0.688

*ASA* American Society of Anesthesiologists score, *CI* confidence interval, *HR* Hazard ratio, *OS* overall survival, *PM* peritoneal metastases

## Discussion

This population-based study aimed to assess the impact of open or laparoscopic approach for CRC on the development of metachronous peritoneal metastases. Patients who underwent open resection of the primary tumour had a significantly higher risk of developing metachronous CPM than patients who underwent laparoscopic resection. This finding contributes to the growing support of the laparoscopic approach given its superior short-term outcomes (i.e. shorter hospital stay, lower complication rate, lower mortality, less major morbidity [10, 11]).

Previously, we reported a lower rate of *synchronous* CPM detected during laparoscopic resection than during open resection [8]. It was hypothesized that the limited overview of the entire peritoneal cavity and the lack of tactile feedback during laparoscopic surgery increased the risk of overlooking peritoneal deposits, resulting in a lower rate of CPM diagnosed during surgery. Eventually, after being overlooked during primary laparoscopic resection, this would subsequently have to lead to a greater number of patients diagnosed with ‘metachronous’ CPM. This phenomenon would be similar to that of the surgical assessment of the peritoneal cancer index, which is also often underestimated during laparoscopic surgery as compared to open surgery [12]. However, this hypothesis was not confirmed by the current study. Instead, the opposite appeared to be true with patients undergoing laparoscopic resection of primary CRC being less frequently diagnosed with metachronous CPM than those who underwent open resection of primary CRC.

The explanation for this phenomenon remains to be elucidated. A possibility may be a difference in surgical trauma as open surgery is known to result in a larger trauma and subsequently a more pronounced pro-inflammatory response [13, 14]. This may result in higher levels of cytokines and growth factors intraperitoneally which may promote the survival and outgrowth of spilled malignant cells into peritoneal metastases. Another reason might be that the embryological planes of dissection are better preserved with subsequent less tumour spill in laparoscopic resection.

However, the differences may also be caused by patient selection. Indeed, patients who underwent open resection more frequently had a T4 tumour stage, nodal involvement, and poorer tumour differentiation. After multivariable regression analyses for these confounders, open resection was still associated with a significantly higher incidence of metachronous CPM. Nevertheless, residual confounding may be present since not all variables that express a poorer tumour biology (e.g. KRAS and/or BRAF mutations, presence of vascular invasion) or factors that complicate laparoscopic surgery (e.g. abdominal wall involvement, acute setting, colonic obstruction) were included in the current analyses, as these were not available for the majority of patients. Adding these factors to the analyses could increase the accurateness of the multivariable model.

The current finding that open resection is associated with an increased incidence of metachronous CPM should not be taken as an argument that all primary CRC resections should be performed by a laparoscopic approach, as it remains unclear whether the surgical approach itself is causing the difference in the incidence of metachronous CPM. In several clinical situations, an open approach may still be preferred, such as an acute setting, colon perforation, T4 tumour, or a history of extensive abdominal surgery [15].

Besides the identification of additional risk factors for metachronous CPM, research should also focus on its prevention. In theory, adjuvant (intraperitoneal) chemotherapy could reduce the risk of metachronous CPM. Nevertheless, two randomized controlled trials were not able to detect a clinical benefit of adjuvant, mainly oxaliplatin-based, intraperitoneal chemotherapy [16, 17]. However, this could also be related to the choice of cytostatic agent, since peritoneal metastases predominantly consist of the consensus molecular subtype 4 (CMS-4), which is considered generally resistant to oxaliplatin [18–20]. The introduction of CPM-derived organoids could allow for a personalized selection of adjuvant (intraperitoneal) chemotherapy [21], aiming to prevent the development of metachronous CPM or to improve their treatment if they develop despite adjuvant therapy.

A limitation of the current study is that residual confounding may still be present because some variables (e.g. KRAS and/or BRAF mutations, presence of vascular invasion) were not available from the NCR. Future studies should focus on the impact of these potentially prognostic factors.

This study also has several merits; it is the first large population-based cohort to investigate the impact of open versus laparoscopic approach on the incidence of metachronous CPM. Also, the NCR is characterized by highly accurate and complete data registration rates, contributing to the interpretability of the results [22]. Finally, all patients in the current cohort were diagnosed in 2015 and thus treated according to the same national guideline for CRC, reducing the chance of bias due to changes in recommended treatments over time.

Results of the current study add further insight into the factors being associated with the development of metachronous CPM. Combined, these can further assist health care providers to select patients who might benefit from intensified follow-up or adjuvant treatment, aiming to reduce the development of metachronous CPM and to increase its detection in an early stage.

In conclusion, patients treated with open resection had a significantly higher risk to develop metachronous CPM than patients treated with laparoscopic resection. The mechanisms underlying this phenomenon remain to be elucidated. However, this finding may further contribute to the development of a personalized follow-up and treatment of patients after primary resection of CRC, aiming to reduce the development of metachronous peritoneal metastases or to detect and treat it as early as possible.
